# Long-term complete remission of metastatic combined hepatocellular-cholangiocarcinoma with nivolumab following failure of targeted therapies: a case report

**DOI:** 10.3389/fonc.2026.1515511

**Published:** 2026-04-14

**Authors:** Young Mi Hong

**Affiliations:** 1 Department of Internal Medicine, Pusan National University School of Medicine, Yangsan, Republic of Korea; 2 Liver Center, Pusan National University Yangsan Hospital, Yangsan, Republic of Korea

**Keywords:** combined hepatocellular-cholanciocarcinoma (cHCC-CCA), immunotherapy, metastatic cancer, nivolumab, targeted therapy resistance

## Abstract

Combined hepatocellular-cholangiocarcinoma (cHCC-CCA) is a rare and aggressive primary liver malignancy with limited treatment options and poor prognosis. We report the case of 33-year-old male with chronic hepatitis B who underwent hepatectomy and pulmonary metastasectomy for HCC with lung metastases. Histological examination revealed cHCC-CCA. However, new lung and chest wall metastases emerged and sorafenib and regorafenib was administered and failed to control tumor progression. The patient was then treated with nivolumab, which led to a complete remission (CR) after 37 weeks. Remarkably, this response has been sustained for over six years without any immune-related adverse events. This case represents a rare case of long-term CR in metastatic cHCC-CCA following treatment with nivolumab after failure of tyrosine kinase inhibitors, suggesting that nivolumab may represent a promising therapeutic option for this challenging malignancy.

## Introduction

Combined hepatocellular-cholangiocarcinoma (cHCC-CCA) is a rare primary liver tumor that exhibits histological features of both hepatocellular carcinoma (HCC) and cholangiocarcinoma (CCA) (1, 2). While surgical resection remains the only curative treatment option for resectable tumors, there is currently no established standare systemic therapy for advanced cHCC-CCA. Systemic therapies are often based on regimens used for HCC and CCA, such as tyrosine kinase inhibitors (TKIs) and gemcitabine combined with platinum-based chemotherapy are commonly used (3). However, the clinical evidence supporting these treatments is limited due to insufficient data (4–8). Only few studies have reported the therapeutic efficacy of immune checkpoint inhibitors (ICIs) in patients with unresectable, inoperable, or metastatic cHCC-CCA (9–11). Here, we present a rare case of metastatic cHCC-CCA in a patient who achieved and sustained complete remission (CR) for nearly six years following nivolumab treatment after the failure of TKIs therapy.

## Case report

**Figure 1:**
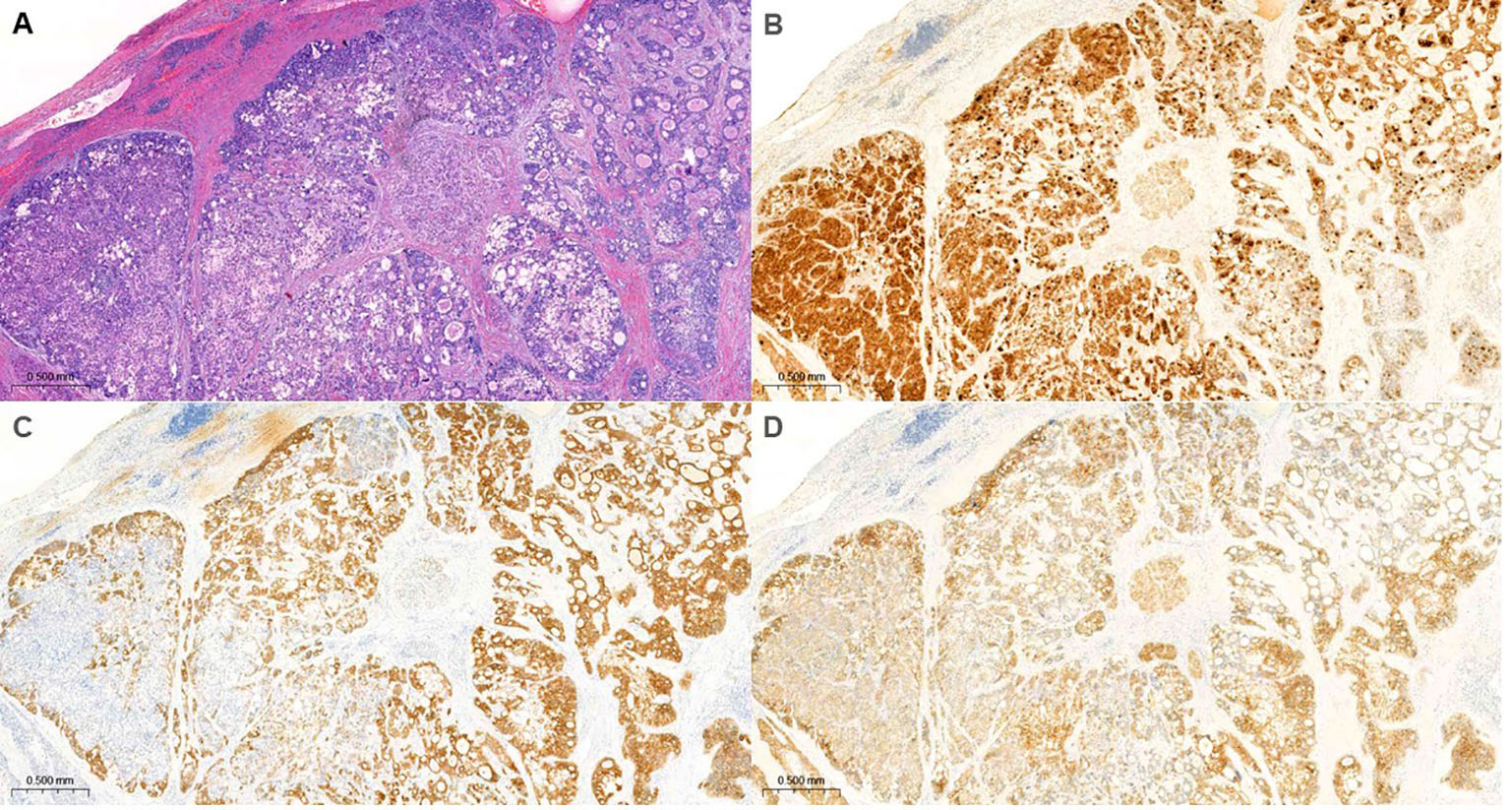
Histopathological and immunohistochemical findings of the tumor demonstrating dual differentiation (20x magnification for all panels). **(A)** H&E stain showing tumor clusters divided by thick fibrous bands. Tumor cluster exhibits a central area displaying well-differentiated, classical HCC-like morphology, and a peripheral area with high cellularity and acinar structures, consistent with a CCA-like appearance. **(B)** Immunohistochemical staining for GPC3 (an HCC marker) shows stronger expression in the central HCC-like areas. **(C)** Importantly, K19 and **(D)** EpCAM (CCA markers) are also observed to be co-expressed within these GPC3-positive HCC-like cells, indicating a biphenotypic differentiation.

We report the case of a 33-year-old-male patient with chronic hepatitis B virus infection who visited the hospital complaining of epigastric pain. He presented with upper quadrant tenderness. He had no history of smoking or alcohol consumption. Computed tomography (CT) and magnetic resonance imaging showed a large, bulging mass measuring 10x12cm in the lateral segment of the liver with massive central necrosis, suggestive of HCC with central necrosis. Additionally, two round pulmonary nodules, each measuring less than 1cm in diameter, were identified. Serological tests showed an alpha-fetoprotein (AFP) level of 133,948 ng/mL, a protein level induced by the absence of vitamin K or antagonist-II (PIKVA-II) level exceeding 75,000 mAU/mL, and a CA 19–9 level of 22.1 U/mL. Based on imaging features, tumor marker levels, and the presence of underlying chronic hepatitis B virus infection, the patient was clinically diagnosed with HCC and lung metastases. In November 2016, the patient underwent hepatic resection and pulmonary metastasectomy. Pathological examination of hepatic mass revealed cHCC-CCA. Microscopically, the tumor was composed of an intimately intermingled population of poorly differentiated HCC and moderately differentiated CCA components within a single tumor mass. Tumor cells positive for glypican-3 exhibited partial immunoreactivity for keratin 7, keratin 19, and epithelial cell adhesion molecule, supporting the biphenotypic differentiation (**Figure 1**).

**Figure 2:**
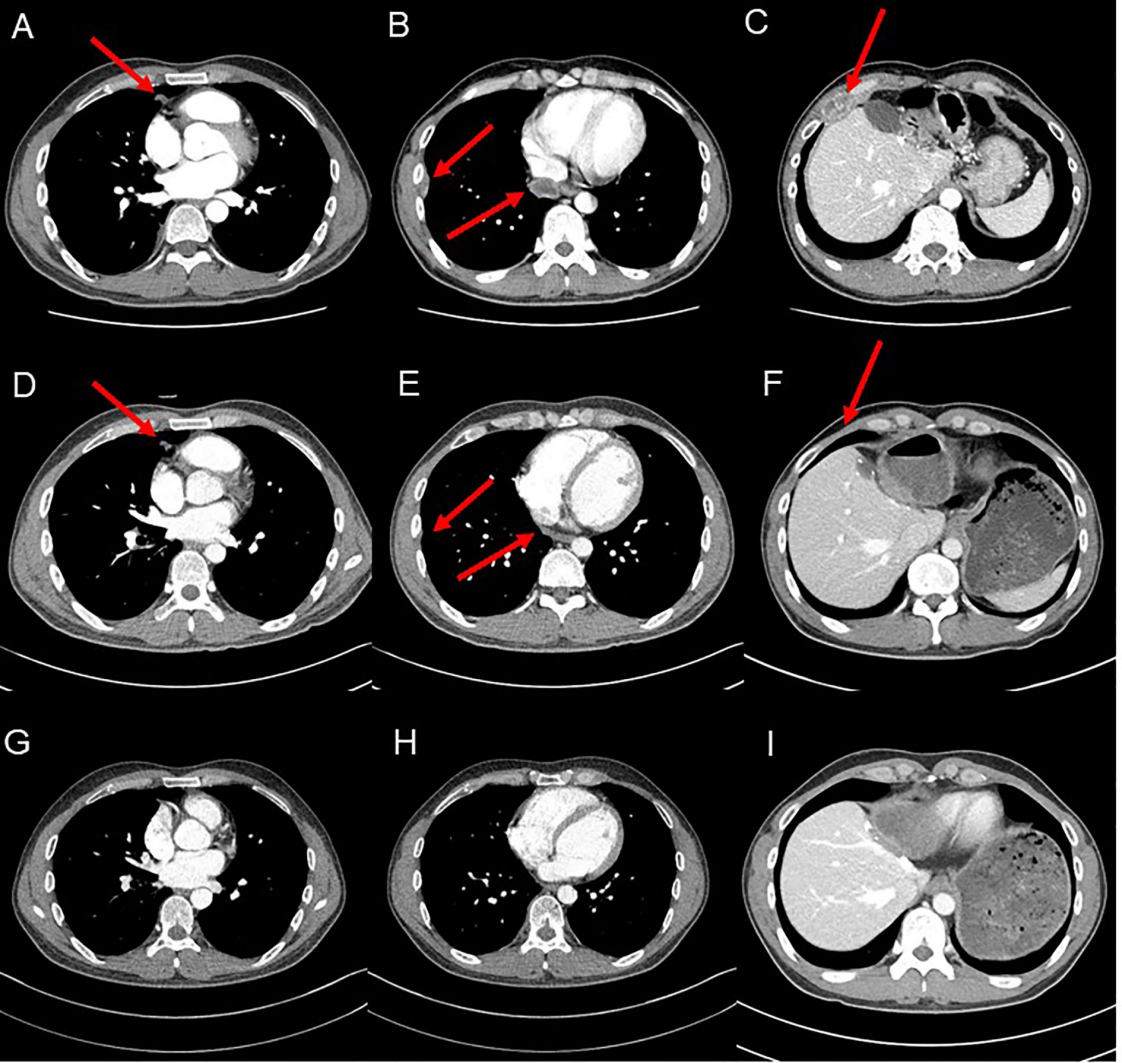
Computed tomography (CT) imaging. CT images showing treatment response to nivolumab. **(A–C)** Baseline CT reveals ovoid hypodense masses (<3.0 cm) at the retrocaval area, right anterior chest wall, and right lateral pleura. **(D–F)** After 9 weeks of nivolumab, the masses show marked size reduction. **(G–I)** After 33 weeks, no definite metastatic lesions are observed in the previously involved sites.

**Figure 3:**
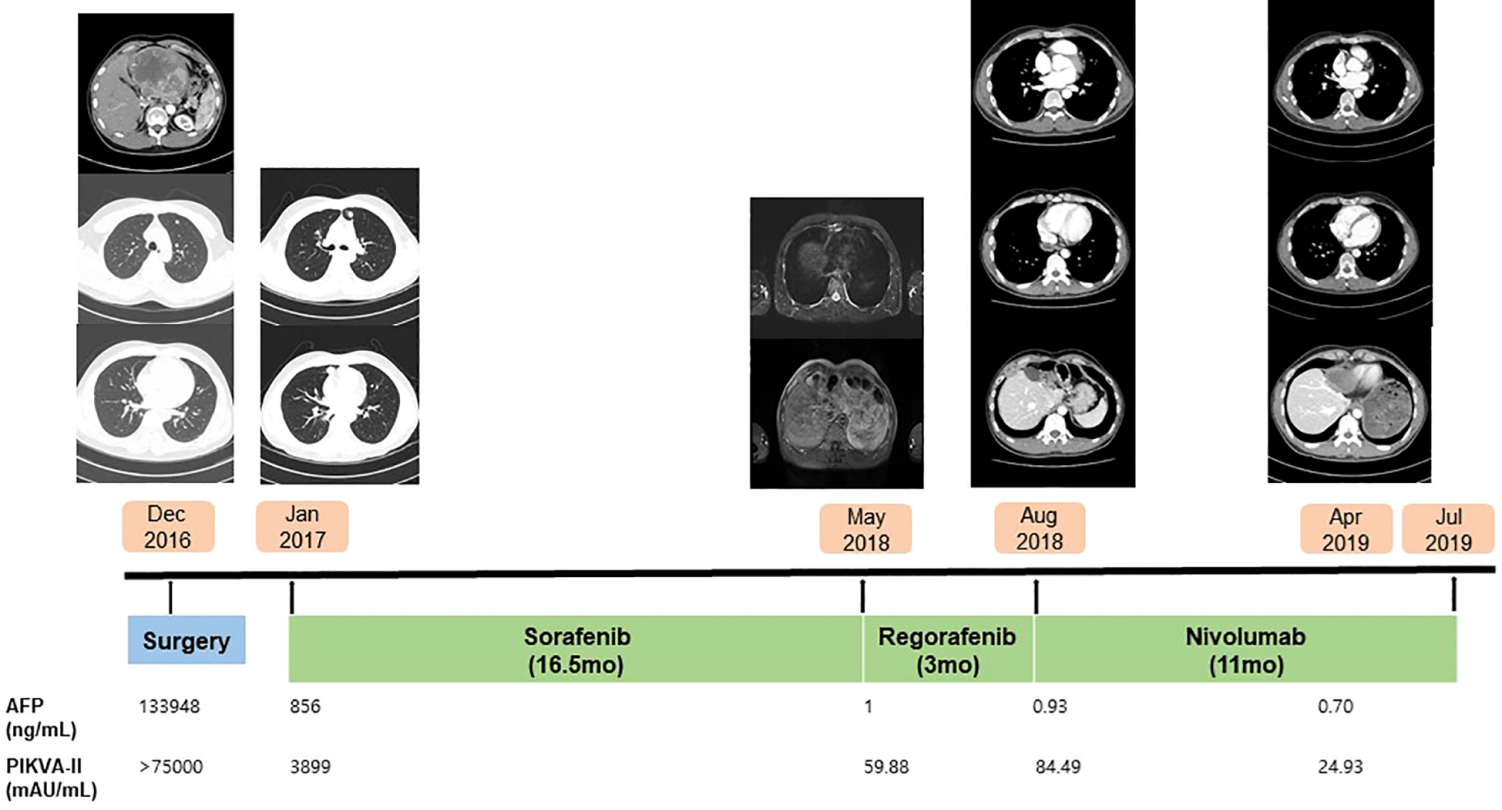
Treatment course. This figure illustrates the chronological sequence of the patient’s treatment, beginning with initial diagnosis in December 2016. It includes hepatic resection and pulmonary metastasectomy, systemic treatment (sorafenib, regorafenib, nivolumab), and the follow-up assessments through July 2019.Changes in AFP and PIVKA-II levels throughout the treatment course are also shown.

Two months after surgery, multiple pulmonary metastases were detected. Sorafenib was initiated as first-line systemic therapy at a dose of 400mg orally twice daily from January 2017 to May 2018. Diarrhea occurred during treatment but was manageable with medication. After 17 months, a partial response was observed, with regression of pulmonary metastases. However, new metastatic HCC lesions, including chest wall involvement, emerged. Regorafenib was then administered as second-line therapy (160mg orally once daily for 3wkks on, followed by 1 week off). The treatment was well-tolerated, with no significant adverse events. Despite three months of regorafenib therapy, the metastatic lesions progressed. CT scan revealed ovoid hypodense masses, less than 3.0cm, in the retrocaval area, right anterior chest wall, and right lateral pleura (**Figures 2A–C**). The patient also reported chest wall pain due to metastasis to the chest wall. At that time, the AFP level was 0.93 ng/mL, and the PIVKA-II level was 84.49 mAU/mL. Consequently, nivolumab therapy was initiated at 3 mg/kg every 2 weeks in August 2018. Follow-up CT scans were performed every 2–3 months. After 9 weeks of treatment, CT scans demonstrated a reduction in metastatic lesions (**Figures 2D–F**). AFP level remained within the normal range, and the PIVKA-II level normalized to 27.34 mAU/mL. The patient’s symptoms improved in parallel with the partial radiologic response. After 33 weeks of nivolumab therapy, complete resolution of metastatic lesions was achieved (**Figures 2G–I**). Nivolumab was discontinued after 24 cycles. The treatment was well tolerated, and no immune-related adverse events or treatment interruptions occurred during therapy. The patient subsequently underwent regular follow-up every 3–6 months with CT imaging and tumor marker monitoring. As of the most recent follow-up in January 2026, no recurrence has been observed on CT, and both AFP and PIVKA-II levels remain within normal range. Remarkably, CR has persisted for more than six years after discontinuation of nivolumab (nine years after surgery). A summary of the patient`s treatment course is presented in **Figure 3**.

## Discussion

cHCC-CCA is a rare and aggressive primary liver cancer that exhibits features of both HCC and CCA. Due to its dual nature, cHCC-CCA presents significant diagnostic and therapeutic challenges, contributing to its generally poor prognosis, which is often worse than that of either HCC or CCA alone (12, 13).

Management of cHCC-CCA remains challenging, as no standard treatment regimen has been established due to its rarity and heterogeneity. Systemic treatment is often based on regimens used for advanced HCC and CCA. Sorafenib, a multi-kinase inhibitor, has been widely used for advanced HCC, while gemcitabine combined with cisplatin has been used for CCA. However, the efficacy of these treatments specifically in cHCC-CCA is not well defined, and data remain limited (14).

In a multicenter study by Gignate et al., outcomes were compared between patients with cHCC-CCA who received either TKIs or platinum-based chemotherapy. The median OS was 8.3 months for the TKIs group and 11.9 months for the chemotherapy group, with no statistically significant difference, suggesting comparable efficacy (8).

The tumor microenvironment plays a crucial role in cancer progression, with immune cell infiltration influencing clinical prognosis. Although a competent immune system can recognize and eliminate malignant cells, cancer cells develop multiple mechanisms to evade the immune surveillance. These include impaired antigen presentation, upregulation of negative regulatory pathways, and recruitment of immunosuppressive cell populations, collectively contributing to immune evasion (15).

The programmed death-1 (PD-1) pathway is a key immune checkpoint exploited by tumors to suppress T cell mediated antirumor response. PD-L1, upregulated on tumor cells, binds to PD-1 receptors on T cells, inhibiting their activity. Nivolumab, a PD‐1 inhibitor, was the first immunotherapy approved for use in HCC. In the CheckMate-040 trial, a sub-group analysis of patients previously treated with sorafenib demonstrated an overall response rate (ORR) of 14%, with a median duration of response of 19.4 months, a disease control rate (DCR) of 55%, and median overall survival (OS) of 15.1 months (16, 17).

Despite several meta-analyses investigating second-line therapies after sorafenib failure, no standard therapy has been established for advanced HCC, primarily due to a lack of head-to-head clinical trials (18–20). In biliary tract cancers, a phase II study of nivolumab in previously treated patients demonstrated an ORR of 22% and a median OS of approximately 14.2 months. The safety profile of nivolumab was consistent with that observed in other cancers (21).

Currently, several combination therapies incorporating PD-1/PD-L1 inhibitors and anti- vascular endothelial growth factor (VEGF) antibodies are under investigation for advanced HCC and CCA, with promising preliminary results (22). Given that demonstrated efficacy of ICIs in both HCC and CCA, immunotherapy may also represent a viable treatment option for cHCC-CCA. However, patient with cHCC-CCA patients are often excluded from most clinical trials, resulting in limited data regarding the efficacy of ICIs in this rare malignancy.

To date, only a few studies, including retrospective analyses and case reports, have evaluated the use of ICIs in unresectable, inoperable, or metastatic cHCC-CCA (9). In a retrospective study of 25 patients who received ICI-based systemic therapy for cHCC-CCA including nivolumab, pembrolizumab, atezolizumab plus bevacixumab, and ipilimumab plus nivolumab, the ORR was 20.0%, with a median duration of response of 11.6 months (95% CI 11.2-12.0 months) (10).Another multicentric retrospective study of 16 patients treated with atezolizumab reported an ORR of 33% (11). Additionally, there were two case reports have described CR following ICI therapy (23). One report presented a patient with metastatic cHCC‐CCA who achieved a durable complete radiological response to pembrolizumab as a third‐line treatment after sorafenib and regorafenib, with the persisting beyond 18 months (24). The second case describes a 67-year-old man with cHCC-CCA who showed an exceptional clinical, radiologic, and biochemical response to ipilimumab plus nivolumab immunotherapy, including near complete response on imaging, and normalization of tumor markers and liver function (25).

The mechanisms underlying the exceptional response in our patient remain unclear but may be attributed to several potential factors. The biphenotypic nature of cHCC-CCA may produce a tumor microenvironment more susceptible to immune checkpoint inhibition. Furthermore, genetic profiling may reveal actionable alterations that enhance sensitivity to ICIs. Prior TKIs treatment may also have modulated the tumor immune microenvironment, enhancing responsiveness to PD-1 inhibitor efficacy. A high mutational burden and strong intrinsic immunogenicity may contribute to immune cell infiltration and antitumor activity. Nivolumab-induced T-cell activation and dynamic shifts in immune phenotype may further potentiate response. However, these hypotheses remain speculative, as molecular and immunohistochemical analyses were limited by the retrospective nature of these studies and the lack of available tissue or genomic data.

Although based on a single case report, our findings provide valuable insights into potential treatment responsiveness of cHCC-CCA to immunotherapy. These results should be interpreted with caution, given the inherent limitations of case reports, including the absence of a control group, inability to establish causality, and lack of molecular validation. Further research and the accumulation of similar cases are warranted to better define the clinical role of immunotherapy in this rare and challenging tumor subtype.

## Conclusion

To the best of our knowledge, this report presents a first case of long-term CR following nivolumab therapy after sequential TKIs failures in a patient with metastatic cHCC-CCA. This case suggests that nivolumab may offer a potential treatment option for selected patients with metastatic cHCC-CCA. However, further clinical studies are needed to validate its efficacy and to identify predictive biomarkers that can guide patient selection and optimize the use of ICIs therapy in this rare and challenging malignancy.

## Data Availability

The original contributions presented in the study are included in the article/supplementary material. Further inquiries can be directed to the corresponding author.
